# QuaBingo: A Prediction System for Protein Quaternary Structure Attributes Using Block Composition

**DOI:** 10.1155/2016/9480276

**Published:** 2016-08-17

**Authors:** Chi-Hua Tung, Chi-Wei Chen, Ren-Chao Guo, Hui-Fuang Ng, Yen-Wei Chu

**Affiliations:** ^1^Department of Bioinformatics, Chung-Hua University, Room S116, No. 707, Section 2, WuFu Road, Hsinchu 30012, Taiwan; ^2^Institute of Genomics and Bioinformatics, National Chung Hsing University, 250 Kuo Kuang Road, Taichung 402, Taiwan; ^3^Department of Computer Science, Universiti Tunku Abdul Rahman, Jalan Universiti, 31900 Kampar, Malaysia; ^4^Biotechnology Center, Agricultural Biotechnology Center, Institute of Molecular Biology, Graduate Institute of Biotechnology, National Chung Hsing University, 250 Kuo Kuang Road, Taichung 402, Taiwan

## Abstract

*Background*. Quaternary structures of proteins are closely relevant to gene regulation, signal transduction, and many other biological functions of proteins. In the current study, a new method based on protein-conserved motif composition in block format for feature extraction is proposed, which is termed block composition.* Results*. The protein quaternary assembly states prediction system which combines blocks with functional domain composition, called QuaBingo, is constructed by three layers of classifiers that can categorize quaternary structural attributes of monomer, homooligomer, and heterooligomer. The building of the first layer classifier uses support vector machines (SVM) based on blocks and functional domains of proteins, and the second layer SVM was utilized to process the outputs of the first layer. Finally, the result is determined by the Random Forest of the third layer. We compared the effectiveness of the combination of block composition, functional domain composition, and pseudoamino acid composition of the model. In the 11 kinds of functional protein families, QuaBingo is 23% of Matthews Correlation Coefficient (MCC) higher than the existing prediction system. The results also revealed the biological characterization of the top five block compositions.* Conclusions*. QuaBingo provides better predictive ability for predicting the quaternary structural attributes of proteins.

## 1. Background

Proteins are responsible for a vast amount of biological synthesis, enzyme catalysis, transport of molecules, and functions in cells. In addition, their specific functions are closely associated with molecular structure. Protein structure can be divided into four levels, that is, from primary to quaternary structure. Many important biological functions must be achieved by polymerization of protein monomers to form oligomeric proteins or higher order multimeric proteins. The concept of protein quaternary structure was first presented by Bernal in 1958 [1, 2], in which he found that some protein compositions and structures were more complicated than others. These proteins were shown to be composed of several protein subunits to form biological macromolecules. The quaternary structures of protein subunits fold together by noncovalent bonds, and thus the structure classification can be delineated according to the type of subunit. If the protein complex consists of identical subunits, it is called a homooligomer; otherwise it is referred to as a heterooligomer. Classification based on the number of subunits can be divided into dimers, trimers, tetramers, and so forth [3]. Examples include (1) insulin, having the activity to form a homodimer; (2) tumor necrosis factor-*α* (tumor necrosis factor-alpha), to form a tight trimer; and (3) human hemoglobin protein is a heterotetramer, with two identical *α* subunits and two identical *β* subunits. An excellent review summarized what is known about the biological functions of nonhomologous homodimer and heterodimeric complexes [4]. For example, thymidylate synthase, a homodimeric protein, is highly conserved among distant species. The tertiary complex of thymidylate synthase has been revealed about the asymmetrical conformation of two homodimers (PDB ID: 4EB4). The closed and open forms of a molecule of the complex dimer may affect the ligand binding strength [5]. In addition, HIV-1 reverse transcriptase is a well-known drug target for treating HIV infections (PDB ID: 3HVT) [6]. Heterodimerization of HIV-1 reverse transcriptase contains subunit P66 and P51 is required for DNA polymerase activity.

Although there has been significant progress in the analysis of protein structure with various experimental approaches, experimentation performed to determine protein structure is typically expensive and time-consuming. Consequently, it is necessary to develop a protein quaternary assembly states prediction system that will enable the analysis of protein structure and function using the current and rapidly increasing amount of sequence data. In previous studies, Garian predicted homodimers and nonhomodimers using a decision-tree and amino acid composition method involving the integration of AAindex. Zhang utilized support vector machines (SVM) and a weighted autocorrelation function in an attempt to identify the key features from the amino acid composition. These studies demonstrated that the primary structure indeed possessed needed information about quaternary structure formation [7, 8]. However, the general feature encoding method of amino acid composition will lose much important protein sequence information, such as physical and chemical properties of amino acids. Therefore, pseudoamino acid composition (PseAAC) was used to predict quaternary structure. This feature not only incorporates the sequence order effect but also reflects hydrophobic and hydrophilic properties [9]. Zhang et al. used PseAAC to develop sequence-segmented PseAAC and combined segments of the protein sequence and domain relationships in an effort to improve prediction results [10]. In recent years, functional domain composition was presented from an evolutionary and functional perspective, because proteins that share similar domain structures often have similar functions [11–13]. This method is suitable for applications in multiple categories of quaternary structural classification problems and can greatly improve prediction performance. However, a disadvantage is that some proteins may not contain any other known functional domains. In fact, the corresponding known functional domains are too few to represent proteins, which result in a classifier being unable to learn effectively. These problems are due to the current database still being incomplete.

The objective of this study is to construct an accurate prediction system for protein quaternary structure attributes. In addition to the previous studies, which have been shown to achieve high prediction accuracy of functional domain composition, the method of functional domains possesses problems that need to be overcome. Accordingly, we attempt to improve this feature extraction method based on a protein sequence homology region concept, that is, block composition, which was proposed to present the protein characteristics. Since the protein interaction binding sites usually have more surface area and a high exposure of hydrophobic solvent accessibility, we will combine amino acid solvent accessibility information and pseudoamino acid composition to calculate the sequence order effect. This system is a three-layer prediction classifier framework. The first layer classifier identifies the structure type of the unknown protein sequence which is, respectively, monomer, dimer, trimer, tetramer, pentamer, hexamer, octamer, decamer, and dodecamer. Then, the result of the first layer of each class serves as input for the second layer classifier, which is used to integrate different features, considering different protein features in the predictive ability of the corresponding advantages and disadvantages to enhance the accuracy of prediction. Finally, the third layer classifier determines the structure type of the query protein.

Cross-validation results show that the predicted results using block composition obtain the best results. Specifically, the overall average prediction accuracy rate is more than 90% in the 60% sequence similarity of each class. Functional domain composition and PseASA are lower by about 10% and 20%, respectively. The results prove that block composition is able to effectively identify quaternary structure assembly states. In addition, performance analysis of different types of function proteins revealed that QuaBingo exhibits superior predictive ability for enzymes, gene regulation, signal transduction, molecular binding, and other important proteins. An online web server is freely available at http://predictor.nchu.edu.tw/QuaBingo/.

## 2. Methods

### 2.1. Compilation of Datasets

The protein oligomer sequences used in this study come from the 3D Complex [14] protein quaternary structure classification database. This database provides protein structures, structure type, symmetrical patterns, and other pieces of relevant information. We searched homo- and heterooligomers of each class from the 3D Complex, and information regarding the corrected number of subunits was utilized to construct the database. The following steps were performed for processing: (1) remove oligomer sequences with lengths of less than 30 amino acids; (2) remove those sequences containing greater than or equal to three unknown amino acid; and (3) use the CD-HIT [15, 16] to remove redundant sequences in the database, that is, the sequence identity with 60%, for avoiding prediction bias. However, the classes of pentamer, octamer, decamer, and dodecamer used CD-HIT 90% for processing to avoid losing sufficient statistical significance. Finally, the database had 8,444 sequences, named Oli8444. This database was employed as the training dataset of the first and the second layer classifiers. Specifically, there were 3,273 monomers, 3,658 homooligomers, and 1,513 heterooligomers. In addition to monomers, the homo- and heterooligomers have eight individual subcategories, that is, dimer, trimer, tetramer, pentamer, hexamer, octamer, decamer, and dodecamer. Heptamer and undecamer sequences are not used due to little available information. The serial numbers of each category are listed in Supplementary Table S1 in Supplementary Material available online at http://dx.doi.org/10.1155/2016/9480276. In order to obtain more representative types of sequences, the training data in third layer classifier are processed by CD-HIT 40% from the Oli8444 training set to further remove sequences containing one or more types of oligomer and named Oli6926. However, the sequence has too few categories, such as pentamer, octamer, decamer, and dodecamer, and is no longer subject to CD-HIT 40% treatment. The independent test is collected from nonlearning test sequences of Oli6926.

### 2.2. Block Composition (Block)

A motif is a small and highly conserved sequence in the secondary structure, which is usually associated with protein function; there are multiple motifs in proteins. The Blocks database [17–19] is a protein motif database which is based on SWISS-PROT and Prosite to calculate ungapped multiple alignment of protein sequences present in short segments of high sequence similarity blocks. Because this feature extraction method is based on searching the sequence of the Blocks database, the method is termed block composition. The Blocks database currently contains 29,068 protein blocks. *P*_Block_ can be defined as 29,068 dimensional space vectors by (1). If the protein *P* can be compared to the corresponding block *i* in the Blocks database, *B*_*i*_ is 1; otherwise it is 0. The rule is defined by the following equation (2). One has

(1) PBlock=B1B2⋯Bi⋯B29068TT  is  the  transpose  operator,

(2) Bi=1,when  a  hit  is  found  for  P  in  the  Blocks  database0,otherwisei=1,2,…,29068.

### 2.3. Functional Domain Composition (FunD)

Proteins usually consist of one or more functional domains. When the same functional domains are discovered in different proteins, this indicates that they may have the same evolutionary origin and function. Version v3.10 of CDD [20] contains 44,354 protein domains and families and includes several external source databases (Pfam [21], SMART [22], KOG [23], COG [23], PRK [24], and TIGR [25]). We use a conservative threshold with *E*-value <0.01 in order to identify what kinds of functional domains are found for query protein *P*. 44,354 proteins can be expressed as a feature vector *P*_FunD_ dimensional space by (3). If *D*_*i*_ is 1, this means that the *i*th domain in CDD is found for *P*, otherwise it is 0. The rule is defined by (3). One has

(3) PFunD=D1D2⋯Di⋯D44354TT  is  the  transpose  operator,

(4) Di=1,when  a  doman  is  found  for  P  in  CDD0,otherwisei=1,2,…,44354.

### 2.4. Pseudoamino Acid Composition Based on Solvent Accessibility of Amino Acid (PseASA)

Protein quaternary structure formed by interactions between two or more polypeptide chains and the interaction depend on surfaces of amino acids in contact with each other. Recent studies of protein hotspots suggest that solvent accessibility constitutes an important feature of protein interactions [26, 27]. Protein binding sites usually have a more exposed hydrophobic area and higher solvent accessibility. Therefore, we will apply this feature in encoding pseudoamino acid composition [9], named PseASA, to investigate the effect of the relationship between protein interactions and structure on the prediction system. First, the information regarding amino acid solvent accessibility is derived from NetSurfP version 1.1 [28] prediction data and divided into “exposed” or “buried” states. The discontinuous exposure and buried amino acid are linked into exposed protein sequence *P*_*e*_ *E*_1_ *E*_2_ *E*_3_ *E*_4_ *E*_5_ ⋯ *E*_*m*_ and buried protein sequence *P*_*b*_ *B*_1_ *B*_2_ *B*_3_ *B*_4_ *B*_5_ ⋯ *B*_*n*_ (*m* and *n* are the sequence lengths and may change with prediction data of different proteins). PseAAC-Builder [29] was used for feature encoding of pseudoamino acids. However, because of the consideration about overall accuracy of using protein features on Oli8444 dataset, QuaBingo did not use the PseASA feature (see Section 3; Tables 1 and 2).

### 2.5. The Three-Layer Architecture of Classifiers

SVM is generally used as a binary classifier that was initially applied to pattern recognition and other fields [30]. In the past, SVM has been successfully applied in various fields of classification problems, and the predictions of quaternary structure have also been found to achieve good results [8, 10, 31]. LibSVM is utilized in this study, and was developed by Chang and Lin [32].

The construction of the prediction system in the current study employs a three-tier architecture, the first layer of which uses SVM to create different characteristic rules of binary classification prediction model. Feature selection using python syntax written LibSVM package fselect.py [33] gives *F*-score based on the importance of each feature and then sorts the trained model by *F*-score. In order to avoid poor recognition and enormous computational time, the trained models are divided into four equal parts according to the *F*-score from high to low and remove 25% or less or more than 75% of the models. Finally, the construction of first layer classification model is completed by choosing better sensitivity, specificity, and Matthews Correlation Coefficient (MCC) based on 10-fold cross-validation accuracy of measurement. Due to 10-fold cross-validation results of first layer classification model, the predictive power of three kinds of characteristic rules for different classes of oligomers was known.

The second layer is the first layer using SVM optimization model predictions, the purpose of which is combining the individual features of each oligomer model outputs into one. Training the second layer integrated model approach is using 10-fold cross-validation test predictions of first layer as input and considering the strengths and weaknesses of the characteristics of different proteins in order to improve accuracy of prediction.

**Figure 1:**
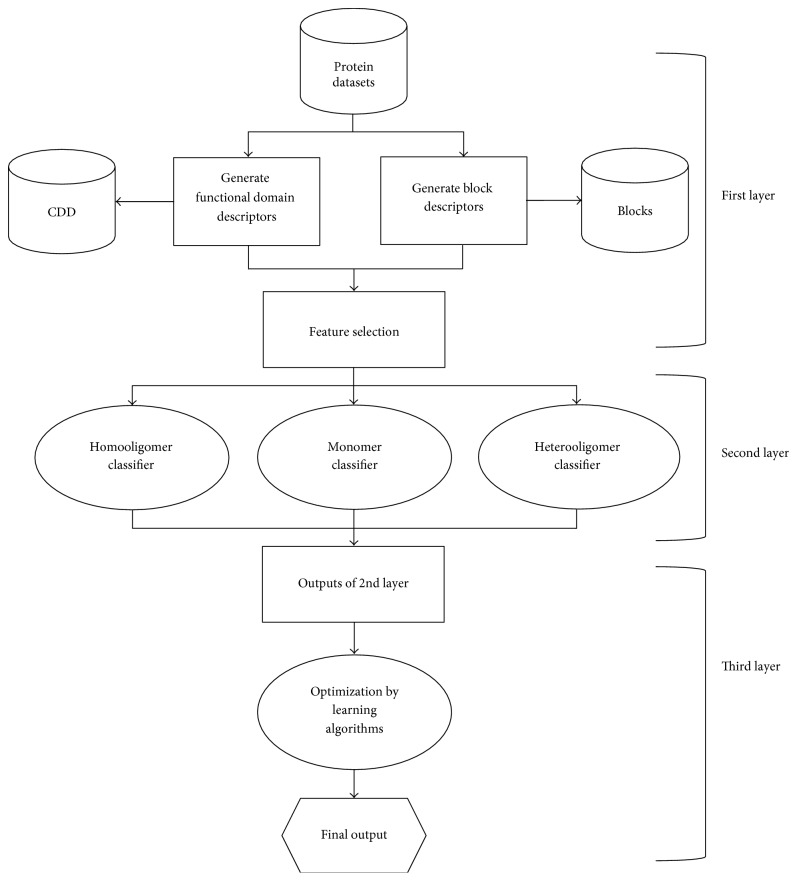
Flowchart of the three-layer architecture of classifiers.

By comparing the data analysis ability of different machine learning algorithms, we finally selected Random Forest to construct the third layer classifier for the integration of these recognition results and determine the quaternary structure type of protein oligomer.  Figure 1 is a flowchart of the predicting system.

### 2.6. Evaluation Measures

To assess the predictive performance of the classifier, we use the following formula. TP, FP, FN, and TN are true positives, false positives, false negatives, and true negatives, respectively. Sensitivity (Sn) on behalf of this type of protein oligomer reflects the percentage of correct predictions for that category. Specificity (Sp) on behalf of nonprotein oligomers of this type indicates the percentage of correct predictions of nonclass. Accuracy (ACC) is used to assess the overall predictive power of the prediction accuracy. Matthews Correlation Coefficient (MCC) values range from −1 to 1, in which the value of 1 represents a completely correct prediction, the value of 0 represents random prediction, and the value of −1 represents exactly the opposite prediction:

(5) Sn=TPTP+FN×100,Sp=TNTN+FP×100%,ACC=TP+TNTP+FP+TN+FN×100%,MCC=TP×TN−FN×FPTP+FN×TN+FP×TP+FP×TN+FN,Recall=TPTP+FN,Precision=TPTP+FP,F-measure=2×precision×recallprecision+recall.

For the third layer classifier evaluation criteria for the classification results, we used Kappa statistics and *F*-measure for viewing. Kappa statistics [34] are used to judge the classifier results, consistent with the random assortment. Its value is in the range of −1 to 1. When *K* = 1, it represents that the predicting results are different with random classifier prediction; *K* = 0 means predicting results are the same as random prediction; *K* = −1 represents that there is no effect and classification credibility. Here, we also use *F*-measure as the evaluation results of the standard classification. *F*-measure is a combination of precision and recall, with values from 0 to 1.

## 3. Results

### 3.1. Performance of Using Different Protein Features in the First Layer

In order to understand the different types of feature codes for the accuracy of the prediction structure, we trained the SVM classification model with 10-fold cross-validation evaluation model validity. Tables 1 and 2 show the 10-fold cross-validation prediction sensitivity, specificity, accuracy, and MCC on the monomer, homooligomer, and heterooligomer.

As can be seen from the results of the cross-validation, block composition in the monomer, homooligomer, and heterooligomer achieved an overall accuracy of 78.91%, 92.27%, and 91.13%, respectively. MCC was 0.579, 0.848, and 0.827, respectively. Since most of sensitivity performance has more than 80%, it indicates that a block composition method is indeed suitable for exhibition of protein characteristics and effectiveness of structure type classification. In the verification results of Functional domains (FunD) feature, the overall accuracy of monomer, homooligomer, and heterooligomer was 75.75%, 80.26%, and 79.93%, respectively. The results of FunD in homooligomer and heterooligomer were lower than the ones of block composition about 10%, while the sensitivity of homooctamer, heterooctamer, and heterodecamer are less than 50%. These results represent that FunD cannot be rendered for associated characteristics. The overall PseASA prediction accuracy is relatively low, that is, respectively, 68.40% and 73.05%. However, compared with the FunD, using PseASA method to predict heterooligomer, pentamer, octamer, and decamer is better at 86.67%, 86%, and 90% of sensitivity, respectively. In addition, the MCC of PseASA for prediction is generally lower, showing that the homology between the whole sequences is not high or that the same category of the sequence number and complexity increases, which makes it difficult to obtain correct predictions. Even if it does not contain pentamer, octamer, decamer, and dodecamer which have a high sequence homology, the overall accuracy of homo- and heterooligomers still reached 90.72% and 90.48%, respectively. To further enhance prediction accuracy, we used the second layer SVM to integrate the various features of the model output.

### 3.2. Performance of Model Combination to Enhance Oligomer Type Prediction Accuracy

The purpose of establishing the second layer is to integrate different predicted results of characteristic model in each category. We unitized different combinations of characteristic models, in which the model is constructed by three features referred to as *B* (Block), *F* (FunD), and *P* (PseASA).  Table 3 displays that performance comparison of model combination in 10-fold cross-validation for oligomer classification in the second layer.

In the result of the monomer combination *B* + *P* with an accuracy of 78.91%, a difference of the combination of *F* + *P* is about 3%. Using a combination of *B* + *F* enhanced accuracy, improving from 78.91% to 82.64%. However, *B* + *F* and *B* + *F* + *P* combination exhibited less accuracy. The same situation also appears in the feature models combination for homo- and heterooligomers. Overall, *B* + *F* model combinations can have better performance than using the single Block model. Most of the categories were improved from 1 to 6%. Therefore, this study will feature *B* + *F* combination to construct the first layer and the second layer of the classification model.

### 3.3. Performance Comparison of Classification Algorithms in the Third Layer

In order to obtain unique results to determine an unknown protein quaternary structure type, we use a layer of the classifier to process the output of the second layer. By comparing different types of algorithms on power of data analysis and problem solving ability, we selected the better algorithm for constructing the third layer classifier. Studies using six types of typical algorithms are tested, that is, Bayes, Functions, Lazy, Rules, Trees, and Meta. The Oli6926 dataset is used in this training. We also used the two authentication methods, 10-fold cross-validation and self-consistency, to assess the learning effectiveness of the classifier.

In the results of 10-fold cross-validation, Correctly Classified Instances (CCI) of LibSVM and Logistic were 67.40% and 67.28%, respectively (Table 4). Kappa statistics was 0.5288 and 0.5285, respectively. And the *F*-measure was 0.616 and 0.615, respectively. These two algorithms have best predicted results. However, we found that the predictive accuracy and statistical value of LibSVM and Logistic are higher because most correct predictions which occurred in the large quaternary categories and in minor categories predictions, like pentamer, hexamer, and octamer, are completely ignored. Other algorithms, such as decision table and Bagging, also have a similar situation. Conversely, the accuracy of Random Forest, Random Tree, and IBk was 58.91%, 54.65%, and 58.45%, respectively. Kappa was 0.4306, 0.3817, and 0.4126, respectively. *F*-measure was 0.566, 0.537, and 0.551, respectively. Although the results of these three algorithms are not perfect, they are not susceptible to imbalance of data numbers.

The results of 10-fold cross-validation of LibSVM and Logistic in the self-consistency test were not significantly increased. Relative under the self-consistency verification, Random Forest, Random Tree, and IBk correctly predicted ratio reached about 90%, since they have good recognition capability for the known information. The prediction performance of Random Forest and IBk was similar in self-consistency which could achieve the highest value of 0.856 MCC. Since the cross-validation and prediction results of Random Forest algorithms for minor categories were good, we finally chose the Random Forest classification algorithm as the third layer classifier in QuaBingo.

### 3.4. Performance Analysis

In order to understand the prediction capabilities of QuaBingo for different functional protein structures in the cell, we compared it with a known quaternary structure prediction tool QuatIdent [12] using an independent test. As shown in Table 5, the predicted result of the average sensitivity of QuaBingo was 51.95%. For the protein categories in the enzyme, gene regulation, membrane protein, single transduction, and molecular binding, there was better prediction of ACC from 77% to 80%. In the QuatIdent, the average sensitivity was 20.74%. These results illustrated the predicting method which is composed of functional domain and PsePSSM cannot obtain a correct identification result for most quaternary protein structures.

### 3.5. The Top Five Features of Block Composition of Oligomer on Oli8444

The feature extraction method of block composition is simple, which implies that a lot of useful information can be gained to help discover mechanisms of protein aggregation and serial modes. We will optimize block composition by feature selection, according to the degree of importance of each characteristic value, giving an *F*-score numerical score. The top five features are shown in Table 6. For example, the IPB006052A of block composition in the top five features is TNF (Tumor Necrosis Factor) family of conserved sequence, which is found in trimeric CD40 ligand (PDB ID: 1ALY) in the training data and also found in the human Collagen X sequences (PDB ID: 1GR3). Human Collagen X needs to rely on the C1q domain to form a stable homotrimer. In existing data annotation, C1q and TNF-like domains overlap, and there are a number of important positions on the sequence of amino acids with high conservation and similar topology [35]. Much literature has confirmed that these amino acids play an important role in the formation of a hydrophobic core stability trimeric structure and formation of biologically active protein complexes [27, 35, 36]. In addition, many other features of block composition are associated with a particular function of protein. Thus, feature selection not only reduces the number of features in block composition but also can effectively identify characteristic patterns obviously related to the protein molecule aggregation phenomenon and hence distinguish quaternary structure among different oligomers.

### 3.6. Case Study

Thymidylate synthase (TS; EC 2.1.1.45) is an enzyme that can converts deoxyuridine monophosphate into deoxythymidine monophosphate and has an important position for necessary cell function about DNA replication and damage repair. The inhibition of TS is a way of cancer treatment that involves using inhibitors to interfere with DNA biosynthesis and create a disturbance in growth of cancer. TS is known that conserved protein from* E. coli* to human. Here, QuaBingo provides the testing results for several TS homologs, including 2KCE (*E. coli*), 4IQQ (*C. elegans*), 2TSR (rat), 4EB4 (mouse), 1HVY (human), and 1I00 (human). The testing results show that QuaBingo can correctly predict the quaternary structure, as homodimer, with TS phylogenetic distant homologs, and the sensitivity performance was 100%. This demonstrates that the QuaBingo may work within the example of phylogenetic homologous proteins.

## 4. Conclusions

In this study, we propose a feature extraction method based on a block of conserved protein sequence for the classification of protein quaternary structure. This method can overcome the problems of feature extraction encountered by functional domain composition: (1) some proteins may not contain any other known functional domains; and (2) corresponding known functional domains are too few to represent proteins. It is worth noting that the first problem has not yet been encountered in our proposed method, and the second problem was comprehensively solved using QuaBingo. The 10-fold cross-validation results showed that the overall accuracy of block composition of homo- and heterooligomers is 92.27% and 91.13%, respectively. Moreover, they are all 10% higher than the functional domain composition. These results demonstrate that the block composition can extract important and biologically meaningful features and thus enhance the prediction of protein quaternary structure.

Although many proteins exist as monomers, they may interact with another protein to form polymers or may further assemble to become a biologically relevant tetramer or octamer. Currently, most of these problems have not been solved through scientific research or verified by adequate information. In the future, as more and more data are added to pertinent databases, an accurate prediction system could be established that would greatly assist relevant research development. An online web server is freely available at http://predictor.nchu.edu.tw/QuaBingo/.

## Supplementary Material

Table S1. The amount of each protein quaternary structure attribute in different datasets.
Oli8444.zip. Dataset Oli8444.

## Figures and Tables

**Table 1:** Performance of using different features with SVM in 10-fold cross-validation for monomer classification on the Oli8444 dataset.

	Monomeric protein			
	Sn (%)	Sp (%)	ACC (%)	MCC
Block				
Monomer	79.07	78.75	78.91	0.579

FunD				
Monomer	93.68	57.80	75.75	0.552

PseASA				
Monomer	70.15	56.33	63.24	0.268

**Table 2:** Performance of using different features with SVM in 10-fold cross-validation for homo- and heterooligomer classification on the Oli8444 dataset.

	Homooligomer				Heterooligomer			
	Sn (%)	Sp (%)	ACC (%)	MCC	Sn (%)	Sp (%)	ACC (%)	MCC
Block								
Dimer	83.18	82.83	83.00	0.660	66.30	97.00	86.73	**0.697**
Trimer	89.25	99.76	95.48	0.909	83.65	97.50	91.93	**0.835**
Tetramer	75.32	97.53	90.12	0.776	85.03	98.03	92.82	**0.853**
Pentamer	100.00	96.67	98.57	0.973	83.33	95.00	89.17	**0.813**
Hexamer	89.03	98.52	94.27	0.887	82.50	97.00	90.42	**0.812**
Octamer	95.71	72.62	84.17	0.709	90.00	85.33	87.67	**0.782**
Decamer	91.67	100.00	95.83	0.928	100.00	100.00	100.00	**1.000**
Dodecamer	95.50	98.00	96.75	0.941	86.00	94.67	90.33	**0.823**

Overall			92.27	0.848			91.13	**0.827**

FunD								
Dimer	52.49	90.26	71.37	0.462	74.74	90.16	85.01	**0.659**
Trimer	93.73	87.83	90.22	0.806	69.94	88.25	80.87	**0.600**
Tetramer	60.64	96.61	84.62	0.647	71.96	95.08	85.79	**0.706**
Pentamer	75.00	86.67	80.48	0.649	53.33	100.00	76.67	**0.572**
Hexamer	64.94	100.00	84.27	0.712	85.75	83.89	84.86	**0.698**
Octamer	48.81	100.00	74.40	0.566	44.67	100.00	72.33	**0.517**
Decamer	63.33	100.00	81.67	0.691	45.00	100.00	72.50	**0.473**
Dodecamer	63.00	100.00	81.50	0.680	69.00	100.00	84.50	**0.733**

Overall			81.07	0.652			80.32	**0.620**

PseASA								
Dimer	66.95	46.74	56.85	0.140	12.62	93.07	66.18	**0.094**
Trimer	39.16	85.93	66.93	0.288	36.08	82.75	63.98	**0.218**
Tetramer	30.11	91.52	71.05	0.280	33.37	83.49	63.34	**0.194**
Pentamer	64.17	70.00	67.62	0.343	86.67	66.67	76.67	**0.564**
Hexamer	65.76	60.37	62.78	0.262	61.63	80.37	71.85	**0.431**
Octamer	66.90	60.48	63.69	0.285	86.00	71.50	78.75	**0.604**
Decamer	81.67	93.33	87.50	0.785	90.00	85.00	87.50	**0.773**
Dodecamer	73.50	68.00	70.75	0.429	66.83	85.50	76.17	**0.556**

Overall			68.40	0.352			73.05	**0.429**

**Table 3:** Performance comparison of model combination with SVM in 10-fold cross-validation for oligomer classification in the second layer.

	*F* + *P*		*B* + *P*		*B* + *F*		*B* + *F* + *P*	
	ACC (%)	MCC	ACC (%)	MCC	ACC (%)	MCC	ACC (%)	MCC
Monomer	75.75	0.552	78.91	0.579	82.64	0.663	82.64	0.663

Homooligomer								
Dimer	71.01	0.456	83.00	0.660	83.00	0.660	83.00	0.660
Trimer	90.22	0.806	95.48	0.909	95.48	0.909	95.47	0.907
Tetramer	84.21	0.638	90.12	0.776	93.41	0.854	93.41	0.854
Pentamer	80.48	0.649	98.57	0.973	98.57	0.973	98.57	0.973
Hexamer	84.27	0.712	94.27	0.887	96.94	0.939	96.94	0.939
Octamer	74.40	0.566	84.17	0.709	85.60	0.743	84.17	0.709
Decamer	85.83	0.759	95.83	0.928	98.33	0.971	98.33	0.971
Dodecamer	81.50	0.680	96.75	0.941	99.00	0.982	99.00	0.982

*Overall*	*81.49*	*0.658*	*92.27*	*0.848*	*93.79*	*0.879*	*93.61*	*0.874*

Heterooligomer								
Dimer	85.01	0.659	86.73	0.697	88.89	0.767	88.89	0.767
Trimer	80.87	0.600	91.93	0.835	91.93	0.835	92.07	0.836
Tetramer	85.79	0.706	92.82	0.853	94.48	0.889	94.48	0.889
Pentamer	77.50	0.577	89.17	0.799	93.33	0.886	93.33	0.886
Hexamer	84.86	0.698	90.42	0.812	90.42	0.812	93.72	0.875
Octamer	72.67	0.484	87.67	0.782	87.67	0.782	87.67	0.782
Decamer	92.50	0.873	97.50	0.958	100.00	1.000	97.50	0.958
Dodecamer	84.50	0.733	90.33	0.823	95.33	0.916	95.33	0.916

*Overall*	*82.96*	*0.666*	*90.82*	*0.820*	*92.76*	*0.861*	*92.88*	*0.863*

**Table 4:** Performance comparison of classification algorithms in 10-fold cross-validation and self-consistency test.

Algorithms	Test method					
	Cross-validation			Self-consistency		
	CCI (%)	Kappa	*F*-measure	CCI (%)	Kappa	*F*-measure
Bayes						
Bayes net	64.80	0.5017	0.608	65.02	0.5053	0.611
Naïve Bayes	39.91	0	0.228	39.91	0	0.228

Functions						
LibSVM	67.40	0.5288	0.616	68.60	0.5464	0.632
Logistic	67.28	0.5285	0.615	67.57	0.5326	0.619
Multilayer perceptron	64.01	0.4893	0.598	69.97	0.5694	0.657

Lazy						
IB1	51.91	0.3513	0.515	87.18	0.8218	0.869
IBk	58.45	0.4126	0.551	90.38	0.8682	0.902
KStar	62.43	0.463	0.581	88.10	0.8362	0.877

Meta						
AdaBoostM1	59.80	0.3909	0.493	59.80	0.3909	0.493
Bagging	66.27	0.5147	0.605	69.88	0.5671	0.65

Rules						
Conjunctive rule	59.80	0.3909	0.493	59.80	0.3909	0.493
Decision table	66.99	0.5189	0.601	67.22	0.5218	0.601
DTNB	67.12	0.5225	0.606	67.38	0.5268	0.61

Tree						
J48	66.45	0.5161	0.607	69.81	0.5639	0.646
Random forest	58.91	0.4306	0.566	90.02	0.8651	0.899
Random tree	54.65	0.3817	0.537	90.38	0.8682	0.902

^*∗*^CCI is correctly classified instances.

**Table 5:** Comparison of results of different functional categories of proteins on QuaBingo and QuatIdent.

Protein categories	QuaBingo				QuatIdent			
	Sn (%)	Sp (%)	ACC (%)	MCC	Sn (%)	Sp (%)	ACC (%)	MCC
Immunity system	40.46	96.28	68.37	0.367	20.61	96.66	58.64	0.199
Enzyme	57.21	97.33	**77.27**	0.545	38.18	97.98	68.08	0.426
Cell cycle	44.44	96.53	70.49	0.410	14.82	97.80	56.31	0.176
Chaperone	45.95	96.62	71.28	0.426	20.27	98.99	59.63	0.313
Gene regulation	58.36	97.40	**77.88**	0.558	21.75	98.19	59.97	0.276
Transport proteins	57.80	97.36	77.58	0.552	21.67	97.86	59.77	0.258
Single transduction	59.16	97.45	**78.30**	0.566	11.97	98.42	55.19	0.167
Viral protein	42.73	96.42	69.57	0.391	10.00	98.75	54.38	0.156
Membrane protein	57.81	97.36	**77.59**	0.552	16.41	98.49	57.45	0.229
Molecular binding	63.37	97.71	**80.54**	0.611	27.11	98.47	62.79	0.351
Hormone	36.08	96.01	66.04	0.321	28.87	97.29	63.08	0.305
Others	60.03	97.50	78.77	0.575	17.18	98.61	57.89	0.247

Overall	51.95	97.00	74.47	0.490	20.74	98.13	59.43	0.259

**Table 6:** Top five features of block composition of oligomers.

Oligomer type	Top 5 features				
	1	2	3	4	5
Monomer	IPB002225A	IPB002347A	IPB000817A	IPB002347D	IPB013549A

Homooligomer					
Dimer	IPB000817A	IPB004045	IPB013572B	IPB001647	IPB003449A
Trimer	IPB007691D	IPB006052A	IPB006056A	IPB006175A	IPB006175B
Tetramer	IPB002347D	IPB003560D	IPB002198B	IPB002347B	IPB002347E
Pentamer	IPB007334A	IPB001931A	IPB013124E	IPB008681A	IPB012599D
Hexamer	IPB001564C	IPB001753C	IPB001980A	IPB001564A	IPB001564B
Octamer	IPB001354C	IPB013341B	IPB002682	IPB001354A	IPB001354B
Decamer	IPB000866A	IPB000866B	IPB013740	IPB003394A	IPB002587G
Dodecamer	IPB002177A	IPB002177B	IPB008331B	IPB014035B	IPB007664A

Heterooligomer					
Dimer	IPB003026B	IPB008386B	IPB000315A	IPB000219A	IPB012565
Trimer	IPB002353B	IPB012565	IPB003990A	IPB001003B	IPB003026B
Tetramer	IPB003026B	IPB012565	IPB010004A	IPB001664D	IPB002398F
Pentamer	IPB001280E	IPB003484D	IPB012420	IPB004333C	IPB006711D
Hexamer	IPB002919A	IPB003038	IPB008019A	IPB001591A	IPB001762
Octamer	IPB007659A	IPB004977B	IPB006574B	IPB002971G	IPB003539A
Decamer	IPB013124E	IPB002662B	IPB003417A	IPB000732A	IPB000817A
Dodecamer	IPB002682	IPB000353B	IPB001003B	IPB003597B	IPB006217A
