# Supplementary material for: QuaBingo: A Prediction System for Protein Quaternary Structure Attributes Using Block Composition
Source: Biomed Res Int. 2016 Aug 17;2016:9480276. doi: 10.1155/2016/9480276 (PMC5005774; doi:10.1155/2016/9480276)
Supplement: Supplementary file 1 — Table S1. The amount of each protein quaternary structure attribute in different datasets. Oli8444.zip. Dataset Oli8444. [file 9480276.f1.pdf]

Table S1. Training datasets for each layer of QuaBingo

Oligomer types	Oli8444	Oli6926
**Monomer**	3273	2764
**Homo-oligomer**		
**Homodimer**	2330	1975
**Homotrimer**	285	237
**Homotetramer**	648	509
**Homopentamer**	31	31
**Homohexamer**	219	172
**Homooctamer**	69	66
**Homodecamer**	28	27
**Homododecamer**	48	48
**Total $^{\text{homo-oligomer}}$**	3658	3065
**Hetero-oligomer**		
**Heterodimer**	602	446
**Heterotrimer**	269	181
**Heterotetramer**	342	227
**Heteropentamer**	25	19
**Heterohexamer**	164	126
**Heterooctamer**	48	39
**Heterodecamer**	16	15
**Heterododecamer**	47	44
**Total $^{\text{hetero-oligomer}}$**	1513	1097

*Oli8444 was used in the 1st and 2nd layer

*Oli6926 was used in the 3rd layer
